# Surgical Management of Calcified Hydatid Cysts of the Liver

**DOI:** 10.1155/1999/20148

**Published:** 1999-07

**Authors:** J. Prousalidis, E. Tzardinoglou, Ch. Kosmidis, K. Katsohis, O. Aletras

**Affiliations:** 1st Propedeutic Surgical Clinic A.U.T. A.H.E.P.A. Hospital 1st Kyriakidis Street-54624 Thessaloniki Greece

## Abstract

Hydatid disease of the liver is still a major cause of morbidity in Greece. Beside the common complications of rupture and suppuration, calcification of the hepatic cysts represent a not well studied, less frequent and sometimes difficult surgical problem. In the present study 75 cases with calcified symptomatic liver echinococcosis were operated on in the 1st Propedeutic Surgical Clinic between 1964 to 1996. Twenty-eight patients were male and 47 female with ages from 23 to 78 years. The diagnosis was based mainly on the clinical picture and radiological studies. In 5 cases the operative method was cystopericystectomy. We performed evacuation of the cystic cavity and partial pericystectomy and primary closure of the residual cavity in 6 cases, omentoplasty or filling of the residual cavity with a piece of muscle of the diaphragm in 4 cases and external drainage by closed tube, in 60 cases. In 12 of those with drainage, after a period of time, a second operation with easy, removal of most of the calcareous wall plaques was performed. The mortality rate was 2%.

Our results could be considered satisfactory. In the calcified parasitic cysts of the liver the proposed technique is cystopericystectomy. An alternative procedure is pericystectomy and drainage with a “planned” reoperation with a bloodless, due to intervening inflammation, chiseling of the calcification.


*HPB Surgery*, 1999, Vol. 11, pp. 253-259
Reprints available directly from the publisher
Photocopying permitted by license only

© 1999 OPA (Overseas Publishers Association) N.V.
Published by license under
the Harwood Academic Publishers imprint,
part of The Gordon and Breach Publishing Group.
Printed in Malaysia.

### *Clinical Series*

### Surgical Management of Calcified Hydatid Cysts of the Liver

J. PROUSALIDIS*, E. TZARDINOGLOU, CH. KOSMIDIS, K. KATSOHIS and O. ALETRAS

*1st Propedeutic Surgical Clinic A.U.T. A.H.E.P.A. Hospital, 1st Kyriakidis Street - 54624 Thessaloniki, Greece*

*(Received 1 August 1998)*

**Hydatid disease of the liver is still a major cause of morbidity in Greece. Beside the common complications of rupture and suppuration, calcification of the hepatic cysts represent a not well studied, less frequent and sometimes difficult surgical problem. In the present study 75 cases with calcified symptomatic liver echinococcosis were operated on in the 1st Propedeutic Surgical Clinic between 1964 to 1996. Twenty-eight patients were male and 47 female with ages from 23 to 78 years. The diagnosis was based mainly on the clinical picture and radiological studies. In 5 cases the operative method was cystopericystectomy. We performed evacuation of the cystic cavity and partial pericystectomy and primary closure of the residual cavity in 6 cases, omentoplasty or filling of the residual cavity with a piece of muscle of the diaphragm in 4 cases and external drainage by closed tube, in 60 cases. In 12 of those with drainage, after a period of time, a second operation with easy, removal of most of the calcareous wall plaques was performed. The mortality rate was 2%.**

**Our results could be considered satisfactory. In the calcified parasitic cysts of the liver the proposed technique is cystopericystectomy. An alternative procedure is pericystectomy and drainage with a "planned" reoperation with a bloodless, due to intervening inflammation, chiseling of the calcification.**

*Keywords:* Calcified cysts, hydatid diseases

### INTRODUCTION

The management of complicated forms of hepatic echinococcosis is generally difficult [1,2]. Nobody knows for sure if the calcified cyst is responsible for the clinical picture, the question to operate or not, especially in the asymptomatic patient, is not easily answered [3]. Very often, due to their size and the advanced age of the usual patient the definitive operation is dangerous and sometimes a staged intervention becomes necessary [4]. On the basis of these problems we studied our cases with this condition.

### MATERIAL – METHODS

In the 1st Propedeutic Surgical Clinic, in the period 1964–1996 a total of 457 patients have

*Corresponding author. Tel./Fax: (00-30-31) 205-927.

undergone surgical treatment for liver echinococcosis. In 75(16,4%) of these cases well formed calcification was apparent. The characteristics of the patients are shown in Table I.

The symptoms were related to superinfection of the cysts or pressure on adjacent structures (Fig. 1). Twenty case were asymptomatic, discovered by chance during admission for another medical problem. On physical examination there was in 5 cases jaundice and in a 30 cases a palpable abdominal mass (Tab. II).

The diagnosis besides the clinical picture was based on various special tests and ultrasound, radiological or isotopic examination (Tab. III, Figs. 2 and 3). The calcification of the "egg shell", punctuate, reticular or lunar type was found in 20 patients. A less serious speckled calcification was noticed in the remainder of the

TABLE I Calcified cysts of the liver

| | Patients |
|---|---|
| Hydatid cysts of the liver (in total) | 457 |
| Calcified cysts | 75 |
| Men | 28 |
| Women | 47 |
| > 50 years of age | 59 |
| < 50 years of age | 16 |
| Range of age 23–78 years | |

TABLE II Clinical picture

| | No. of cases |
|---|---|
| Septic fever | 25 |
| Upper abdominal pain or discomfort | 25 |
| Loss of weight | 10 |
| Asymptomatic | 20 |
| Palpable mass | 30 |
| Jaundice | 5 |

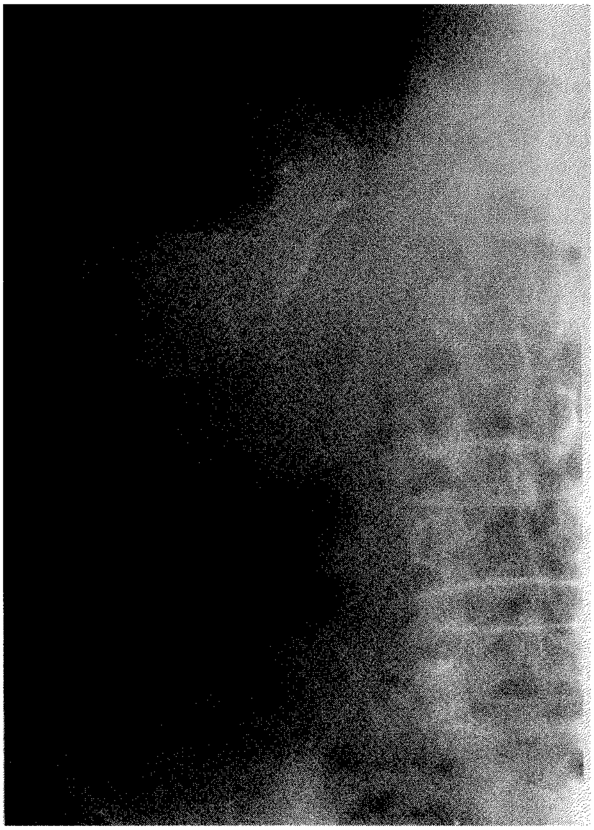

FIGURE 1 Abdominal X-ray. Huge calcified hydatid cyst of the liver with suppuration.

TABLE III Diagnostic examination

| | Patients | Positive |
|---|---|---|
| Casoni | 20 | 10 |
| Complement fixation | 20 | 8 |
| Indirect hemaglutination | 18 | 10 |
| Elisa | 20 | 10 |
| Sonogram | 50 | 50 |
| Abdominal X-ray | 75 | 75 |
| CT scan | 35 | 35 |
| Isotopic scan | 15 | 15 |

cases. The liver isotopic scans with their non specific filling defects or the immunological tests, two or more in combination, played an important diagnostic role mainly in the earlier part of this study.

The surgical approach was abdominal in 49 cases (right or double subcostal, midline), thoracic (8 cases) and right thoracoabdominal (18 cases).

In 50 cases the parasite was single and in 25 cases multiple (Fig. 4). The calcified cyst was located in the right lobe in 63 patients, in the left in 7 and in both lobes in 5 patients. Their size ranged from 7 to 25 cm, and all of them were multivesicular. The content of the cysts was an amorphous yellow or pus like mass with torn, mother or daughter, laminated membrane elements (45 cases) or intact daughter cysts (30 cases).

The operative technique in 5 patients was cystopericystectomy. In the other 70 patients after evacuation of the cystic cavity and partial pericystectomy, primary closure of the residual

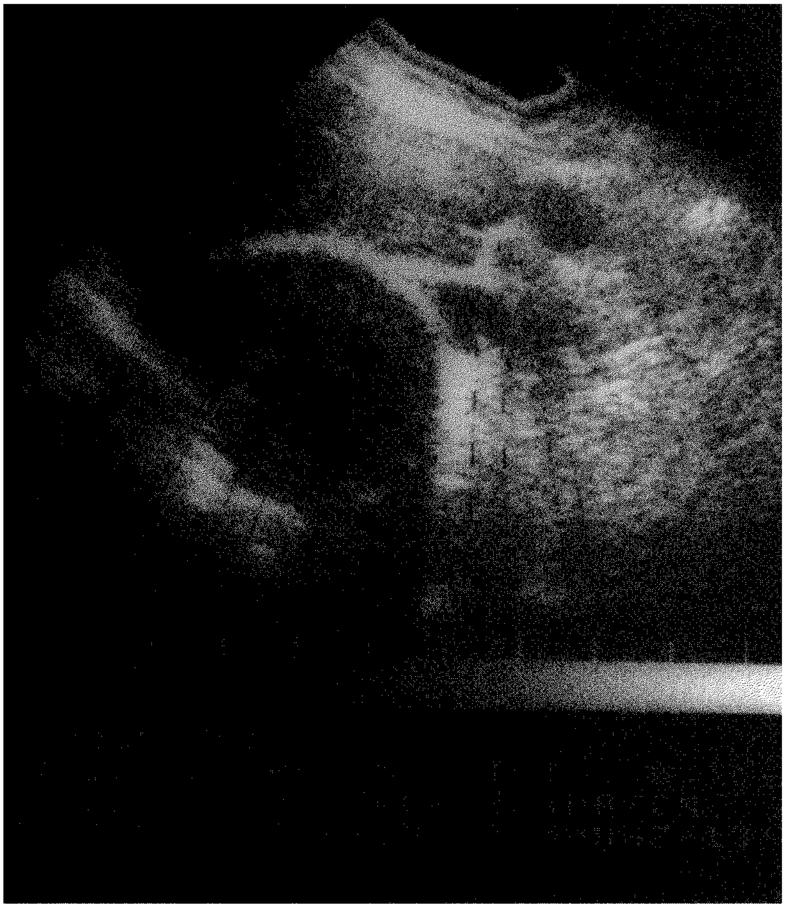

FIGURE 2 Sonogram. Calcified hydatid cyst of the liver.

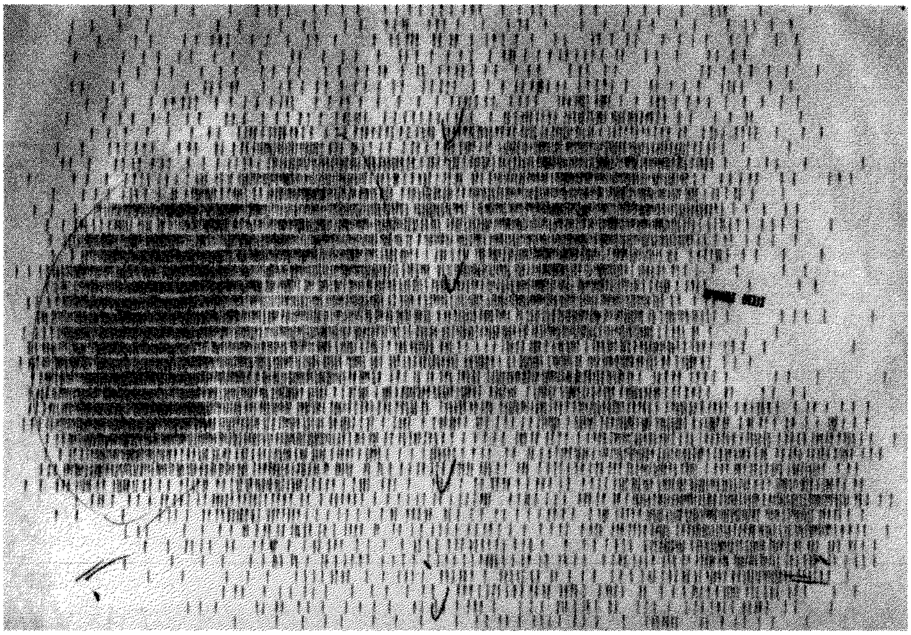

FIGURE 3 Isotopic scan. Calcified hydatid cyst of the liver.

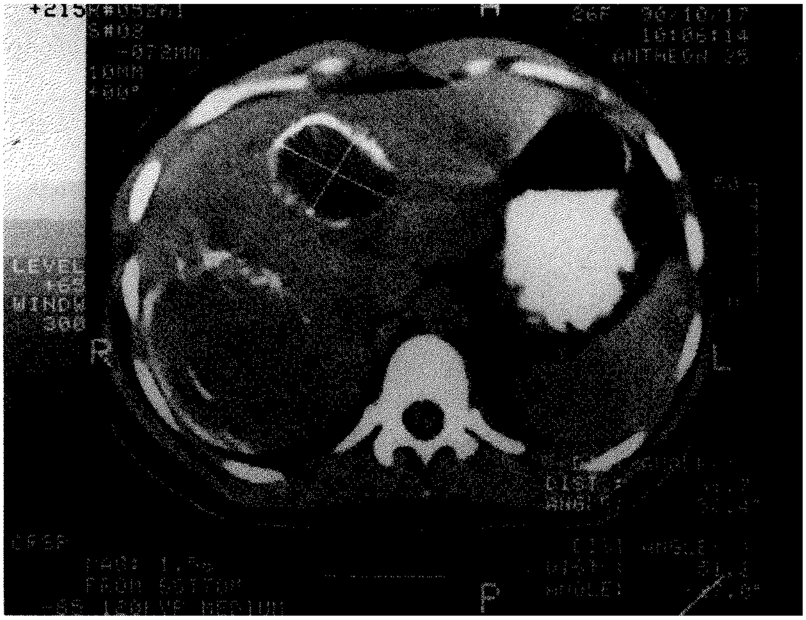

FIGURE 4 Abdominal CT scan. Multiple calcified hydatid cysts of the liver.

cavity (6 cases), omentoplasty or filling of the residual cavity with a piece of muscle of the diaphragm (4 cases), and external drainage by closed tube (60 cases) was done. In 20 of them, with heavy calcification, only a limited removal of the calcified wall of the remaining cavity was attempted. The total chiseling of the remainder of the pericyst was dangerous due to local bleeding and leakage of bile. The wall of the cysts were scrubbed with swabs, dipped in the initial cases into formalin or in the last fifteen years into hypertonic saline. The surgical procedures used are listed in Table IV.

Biliary stones were found in 6 patients. Incidental cholocystectomy in 24 patients with bile duct exploration in 17 of them (positive for stones in 3 cases and for parasitic element in 4) was done. Subphrenic or subhepatic drain was used in all the cases.

### RESULTS

Two patients died. The first patient, with multiple cysts, and chronic obstructive pneumonopathy died in the 5th postoperative day from heart failure. The second patient, with a single cyst, died with sepsis in the 23rd postoperative day due to duodenal ulcer perforation.

The postoperative hospitalization ranged from 12 to 35 days. The average hospital stay in the cases treated with cystopericystectomy, and with partial pericystectomy plus omentoplasty, myoplasty or drainage, was 15 and 28 days respectively.

In 12 patients, with cysts of huge size and stiff, non collapsing walls, treated with pericystectomy and drainage procedure, persisting suppuration of the cystic cavity developed. On account of this complication after a period of 30 to 90 days, a reintervention was decided. During this time, the previously reported difficult calcinous debulking, was technically easy. The plaques were almost completely separated from the pericyst wall, and the remaining cavities were clearly shrunk. In a number of patients other postoperative complications, managed conservatively, were noticed (Tab. V).

In the follow up of the patients 1 to 32 years later, no recurrence was noted.

### DISCUSSION

Calcification of the parasite occurs in about 10% to 16,6% of the cases of hepatic echinococcosis, and it may require about 5 to 10 years to develop, being very common in the aged population [3–6]. In our series the calcified parasitic cysts comprised 16,4% of the total number of hydatid cysts of the liver and the mean age of the patients was also advanced.

The general opinion is that, most of these cysts are biologically and clinically silent. They do not rupture into the bile duct, the thorax or the abdominal cavity and they rarely become infected [3,4,7]. This is not always true. According to other writers and our observations, the calcification does not exclude either clinical or laboratory abnormalities or intrabiliary and intrapulmonary penetration. The parasite in some cases is still viable and the cyst continues to grow even if a portion of its wall has become necrotic and calcified [2,4,8,9]. Some of our patients were almost asymptomatic and the diagnosis was based on plain X-ray film, sonogram or CT scan taken for another purpose, but most of them were admitted with various

TABLE IV Type of operative procedures

| | Patients |
|---|---|
| Cystopericystectomy | 5 |
| Partial pericystectomy, capitonnage | 6 |
| * Partial pericystectomy, drainage | 60 |
| Partial pericystectomy, omentoplasty, myoplasty | 4 |

* Limited chiseling of heavy calcification (20 cases), Cholecystectomy (24 cases) + B.D.E. (17 cases).

TABLE V Postoperative complication

| | Patients |
|---|---|
| Chest problems | 6 |
| Choloperitoneum | 1 |
| Wound infection | 8 |
| * Suppuration | 12 |
| Total | 27 |

* Reoperation with easy calcinous debulking.

symptoms or had developed complicated forms of the disease. Despite the rate of false negative results, due to low antibody titers, the specific intradermal and serologic tests had in some cases a diagnostic role [2, 7].

The calcific salts accumulate mainly in the adventitia or sometimes in the wall of mother or daughter cysts with consequent partial or total calcification. Depending on the extent of the calcification several typical findings in the imaging techniques are described in this and other series [3, 4, 9]. The most characteristic on US, CT, MR (also on plain films) is the round egg shell picture when the pericyst is totally calcified. In these cases the inside demonstration of fertile daughter cysts is impossible with ultrasound but easily done with CT. Computerized tomography is also the method of choice in the detection of microcalcifications in the early phases of the degenerative process [10]. The congregation of calcinous deposits in the content of the cysts, the folded mother or daughter elements, gives a reticular, punctuate or other variation [3, 9]. Scintiscanning, in the space occupying liver lesions, performed in our earlier cases, is not specific and like angiography has been replaced [2, 7]. Endoscopic retrograde cholangiography, diagnostically and for therapeutic reasons, may be indicated, in cases with cholestatic jaundice [11]. It is an alternative method to open exploration of common bile duct.

Differentiation of the calcified hydatid cysts from chronic amoebic abcesses, benign and malignant primary and secondary tumours, or other causes of calcification in the right upper quadrant, rarely nowdays pose problems [1, 3].

In contrast to the easy diagnosis, the management of the calcified parasite is sometimes difficult. Chemotherapy with benzimidazole and other compounds is not, in these cases, indicated due to the stiff cystic wall which has to be penetrated by drugs in order to reach all the compartment of the metacestode [7, 13]. The PAIR method (puncture of cysts percutaneously, aspiration of fluid, introduction of protoscolicidal agent and reaspiration) under sonographic and/or fluoroscopic guindance is another option under evaluation in selected cases [14, 15]. There is no indication in patients with complicated hydatid disease, like these in our study, to perform these types of treatment and the choice of therapy has to be decided between conservative (to leave the cyst alone and the patient to be followed up) or operation [15].

There is the consensus that the small calcified cysts (less 5 cm in diameter) should not be treated surgically [2–4]. For cysts over 5 cm some suggest these are best left untreated if they are silent. But the policy of others and our opinion is that every large cyst should be operated regardless the degree of calcification and the clinical picture [2–4, 8]. It is difficult to persuade oneself and the patient that no complication can come from an asymptomatic but palpaple large liver parasite especially, with fluoroscopically detectable daughter cysts inside as in some of our cases [3, 9]. The exception to this therapeutic strategy is the cyst in an aged patient with concomitant diseases. It has been shown, by others, that there are few fertile cysts in patients older than 60 years and the risks of postoperative morbidity in this age are more than the chances for problems from the presence of the cysts [2, 4, 16]. In general it is out of the question, as we have practiced in our series, not to operate on calcified parasites, when they coexist with other cysts or are complicated or if they produce symptoms due to expansion or great weight [3, 4].

Regarding the incision, we know that it must afford the maximum exposure, especially when tedious dissection for cystopericystectomy is anticipated or in cysts in the liver dome [4]. In most of the cases in this study adequate exposure was obtained by abdominal incision but thoracic or a thoracoabdominal approach was sometimes necessary. The type of surgery, in these cases depends on the cyst characteristics and follows the operative principles used in hepatic hydatidosis [2-4]. Partial atypical or standard liver resection for peripheral, pedunculated or very large calcified cysts rarely is indicated. Cystopericystectomy, when it is possible, is the ideal technique for medium sized cysts, cautiously performed in the plane between the adventitia and the liver parenchyma. This method is performed in a limited number of cases of this study, but avoided in cysts located deeply in the parenchyma, near the inferior vena cava or the liver hilum. The usual procedure, is the complete removal of the content of the cyst, perioperative inactivation of active scolices and partial pericystectomy with, removal of as much as possible of calcareous parts of the adventitia [3,4]. Because in these patients any type of capitonnage is very difficult, the methods of treating the residual cavity, is tube drainage, rarely omentoplasty or sometimes leaving the cavity wide open. Cholecystectomy due to lithiasis, daughter cysts within, or due to nearby cysts encroaching it, is mandatory [3]. Routine performance of this operation when the gallbladder is simply in some proxipity with cysts of the liver is not warranted.

Although complete cure of calcified cysts is achieved, according to the bibliography and our experience the prognosis should be guarded [2-4]. In other and this series the mortality is low, no recurrence is reported, but the conservative operative approach usually performed, is followed by a high morbidity rate [3,17]. Due to non collapsing walls and incomplete removal of calcareous plaques of the cysts, serious bile drainage and secondary infection was sometimes noticed. In the extensively calcified forms of the disease as we have observed in our patients, a supplementary operation is sometimes necessary. This reintervention is almost considered to be a planned or second stage procedure. The chiseling of the remaining calcinous layer of the cyst is, compared with the initial operation, much easier with satisfactory results. The small, peripheral calcified cysts managed usually with radical methods, are followed with lower postoperative complication rate [17]. Some of these cases are being removed completely and safely, under videolaparoscopy, an approach with well known advantages [18]. This new technical possibility may be proved, in the future, as a helpful alternative to open surgery in even more cases of hepatic hydatidosis.

### *References*

[1] Schaefer, J. and Yousuf khan, M. (1991). Echinococcosis (hydatid disease): Lessons from experience with 59 patients. *Rev. Infect. Dis.*, **13**, 243.

[2] Milicevic, M. (1994). Hydatid disease in Blumgart L. H., Surgery of the Liver and Biliary tract. Vol. II, Churchull Livingstone, pp. 1121-1150.

[3] Saidi, F. (1976). Surgery of hydatid disease. Saunders company London-Philadelphia-Toronto.

[4] Kourias, B. C. (1974). Hydatid disease of the liver and other viscera in Maingot. Abdominal operations, Sixth edition, **2**, 1271-1292.

[5] Caratozzola, M., Scardella, L., Grossi, G. *et al.* (1991). Diagnostic approach of abdominal hydatidosis by ultrasonography. *Arch. Hidatid*, **30**, 531.

[6] Reeder, M. M. and Palmer, P. E. S. (1981). The Radiology of Tropical Diseases with Epidemiological, Pathological and Clinical Correlation. Baltimore, Williams and Wilkins.

[7] Ammann, R. W. and Eckert, J. (1996). Cestodes Echinococcus. *Gastroenter. Clin. North Amer.*, **25**, 655.

[8] Pissiotis, C. A., Wander, J. U. and Condon, R. E. (1972). Surgical treatment of hydatid disease. *Archives of Surgery*, **104**, 454.

[9] Lewall, D. and Mc Corkell, S. (1986). Rupture of echonococcal cysts. Diagnosis, classification and clinical implication. *A. J. R.*, **146**, 391.

[10] Franquet, T., Moutes, M., Lecumberri, F., Esparza, J. and Besvos, J. (1990). Hydatid disease of the spleen: Imaging findings in nine patients. *A. J. R.*, **154**, 525.

[11] Grima, N. (1997). Endoscopic management of hydatid disease of the liver. Archivos Internationales de la Hidatidosis **XXXII**, 143.

[12] Caratozzola, M., Scardellce, L., Grossi, G. *et al.* (1991). Diagnostic approach of abdominal hydatidosis by ultrasonography. *Arch. Hidatid*, **30**, 531.

[13] Thompson, R. C. A. and Lymbery, A. J. (1995). Echinococcus and hydatid disease, Wallingford, Oxon/UK, CAB International.

[14] Kammerer, W. S. and Schantz, P. M. (1993). Echinococcal disease. *Infect. Dis. Clin. North Am.*, **7**, 605.

[15] Pelaez, V. (1997). Experience with PAIR in America. Archivos internationales de la Hidatidosis **XXXII**, 159.

[16] Little, J. M. (1976). Hydatid disease at Royal Prince Alfred Hospital 1964 to 1974. *Med. J. of Australia*, **1**, 903.

[17] Safioleas, M., Misianos, E., Manti, Ch., Katsikas, D. and Scalkeas, G. (1994). Diagnostic evaluation and surgical management of hydatid disease of the liver. *World J. Surg.*, **18**, 859.

[18] Saglam, A. (1996). Laparoscopic treatment of liver hydatid cysts. *Surgical Laparoscopy Endoscopy*, **6**, 16.

